# Milder symptoms and shorter course in patients with re-positive COVID-19: A cohort of 180 patients from Northeast China

**DOI:** 10.3389/fmicb.2022.989879

**Published:** 2022-10-11

**Authors:** Hongyan Li, Mingqin Zhu, Peng Zhang, Xingjian Yan, Junqi Niu, Zhenyu Wang, Jie Cao

**Affiliations:** ^1^Department of Nursing, The First Hospital of Jilin University, Changchun, China; ^2^Department of Neurology, The First Hospital of Jilin University, Changchun, China; ^3^Department of Infectious Diseases, The First Hospital of Jilin University, Changchun, China; ^4^Department of Urology, The First Hospital of Jilin University, Changchun, China; ^5^Key Laboratory of Organ Regeneration and Transplantation of the Ministry of Education, State Key Laboratory of Zoonotic Disease, Department of Hepatology, Center of Infectious Disease and Pathogen Biology, The First Hospital of Jilin University, Changchun, China; ^6^Department of Spinal Surgery, The First Hospital of Jilin University, Changchun, China

**Keywords:** COVID-19, re-positive, Northeast China, anti-CoV IgG, underlying disease

## Abstract

China experienced another widespread Coronavirus disease 2019 (COVID-19) outbreak recently caused by the Omicron variant, which is less severe but far more contagious than the other COVID-19 variants, leading local governments to focus efforts on eliminating the spread of the disease. Previous studies showed that after “recovering” from the virus, some patients could re-test positive for COVID-19 with nucleic acid tests, challenging the control of disease spread. In this study, we aimed to analyze the clinical and laboratory characteristics of re-positive COVID-19 patients in Northeast China. We retrospectively analyzed data from confirmed reverse transcription polymerase chain reaction (RT-PCR) re-positive COVID-19 patients who were admitted to the First Hospital of Jilin University, Jilin Province, China, from March to June 2022. Detailed clinical symptoms, medical history, anti-Corona Virus (CoV) IgG and IgM levels, and CoV nucleic acid cycle threshold (Ct) values during the re-positive period were collected and analyzed. A total of 180 patients were included in this study, including 62 asymptomatic cases and 118 mild cases. The cohort included 113 men and 67 women, with an average age of 45.73 years. The median time between recovery from the virus and re-positivity was 13 days. Our results showed that the proportion of re-positive patients with symptoms was lower, and the nucleic acid test-positive duration was shorter during the re-positive period. Furthermore, in patients with underlying disease, the proportion of patients with symptoms was higher, anti-CoV IgG levels were lower, and the total disease duration was longer. In conclusion, during the re-positive period, the symptoms were milder, and the CoV nucleic acid test-positive course was shorter. The concomitant underlying disease is an important factor associated with clinical symptoms, and the overall course of COVID-19 re-positive patients may be associated with lower anti-CoV IgG levels. Large-scale and multicenter studies are recommended to better understand the pathophysiology of recurrence in patients with COVID-19.

## Introduction

Coronavirus disease 2019 (COVID-19), caused by severe acute respiratory syndrome coronavirus-2 (SARS-CoV-2), has had a worldwide impact since its first identified case in 2019. The Omicron variant of SARS-CoV-2, identified in November 2021, has contributed to a recent increase in the number of cases worldwide. This variant is heavily mutated, with a very high risk of infection, making the pandemic more complex and difficult to control (Araf et al., 2022). The pandemic in Changchun, Jilin Province, from early March 2022, was caused by the new highly contagious Omicron mutant BA.2 strain. However, infected individuals have atypical symptoms or are asymptomatic, resulting in the spread of the disease within the community.

Previous studies have shown that re-positive tests for SARS-CoV-2 using nucleic acid reverse transcription polymerase chain reaction (RT-PCR) in recovered COVID-19 patients are common (Simon et al., 2020; Li et al., 2021; Wong et al., 2021). The proportion of re-positive tests in recovered COVID-19 patients varied from 2.4 to 69.2% and ranged from 1 to 111 days after recovery, depending on region, sample size, age of patients, and type of specimen (Chen et al., 2020; Mattiuzzi et al., 2020; Vancsa et al., 2021). However, the mechanisms underlying these positive test results remain unclear. Currently, several causes of re-positive tests for SARS-CoV-2 in recovered COVID-19 patients are proposed, including false-negative RT-PCR tests, prolonged or recurrent viral shedding, reactivation, or reinfection with coronavirus (Miyamae et al., 2020). The false-negative RT-PCR results may be due to the sources of samples. Oropharyngeal and nasopharyngeal (OP/NP) swab samples are often used for testing; however, SARS-CoV-2 has been found in fecal specimens of patients with clinically suspected COVID-19 but multiple negative RT-PCR test results for SARS-CoV-2 using OP/NP specimens (Brogna B. et al., 2021). Sensitivity/specificity problems of the nucleic acid test kit and the sampling procedure itself have also been reported to contribute to the false-negative results. Reinfection needs to be confirmed by viral gene sequencing results or clear epidemiological data; knowledge about reinfected patients remains limited (Farrukh et al., 2021).

Theoretically, re-positive patients can be potential transmission sources. The transmission capacity of recurrent SARS-CoV-2 depends on viral replication. The viral load in re-positive cases is very low, and the viral culture of the OP/NP samples from re-positive patients showed no cytopathic effect (Liang et al., 2021). However, there is evidence showing spontaneous replication of SARS-CoV-2 in the bacterial cultures of patients’ feces for up to 30 days and beyond, indicating that the gut microbiota could affect the severity of COVID-19 (Yeoh et al., 2021). In addition, toxin-like peptides, which were almost identical to the toxic components of animal venoms, such as conotox, phospholipases, phosphodiesterases, zinc-metal proteinases, and bradykinins, were reported in the blood, feces, and urine obtained from patients with COVID-19 (Brogna et al., 2022). The existence of toxin-like peptides has been suggested to be associated with a large set of heterogeneous extra-pulmonary COVID-19 clinical manifestations (Brogna B. et al., 2021). Owing to the potential risk of virus transmission in recovered patients with re-positive virus detection, after discharge, recovered patients were transferred to quarantine locations for another 7 days, and a coronavirus nucleic acid test (COVNAT) was performed during the quarantine period.

In this study, we aimed to analyze the clinical and laboratory characteristics of re-positive COVID-19 patients from Changchun, Jilin Province, and to discuss the factors influencing disease severity in these patients. Our findings will aid in the management of patients with COVID-19 caused by the Omicron variant of SARS-CoV-2.

## Materials and methods

### Study design and participants

Clinical and laboratory data from patients admitted to the First Hospital of Jilin University between March and June 2022 were retrospectively collected. COVID-19 was diagnosed according to the Diagnosis and Treatment Protocol for COVID-19 (Trial Version 9) criteria (National Health Commission & National Administration of Traditional Chinese Medicine, 2022) issued by the National Health Commission of China.

### Ethical statements

The studies involving human participants were reviewed and approved by the Ethics Committee of the First Hospital of Jilin University. Written informed consent for participation was not required for this study in accordance with national legislation and institutional requirements.

### Clinical definition

Coronavirus disease 2019 was diagnosed by measuring SARS-CoV-2 RNA in oropharyngeal swabs. RNA was measured using RT-PCR according to the standard protocol by specialized laboratory personnel at the Changchun Infectious Hospital.

The severity of COVID-19 was defined according to the Diagnosis and Treatment Protocol for COVID-19 (Trial Version 9) criteria: (1) asymptomatic type: COVNAT positive without any clinical symptoms; (2) mild type: mild clinical symptoms without any radiological findings; (3) general type: limited clinical symptoms (i.e., fever, cough, and other common pneumonia-related symptoms with radiological abnormality); (4) severe type: patients with any of the following: (a) respiratory distress, respiratory rate ≥ 30 per min; (b) oxygen saturation on room air at rest ≤ 93%; and (c) partial pressure of oxygen in arterial blood/fraction of inspired oxygen ≤ 300 mmHg; and (5) critical type: patients with any of the following: (a) respiratory failure with mechanical ventilation required; (b) shock; and (c) other organ dysfunction requiring intensive care unit monitoring treatment.

The COVID-19 patients were considered to be discharged when they met all the criteria from the Treatment Protocol for COVID-19 (Trial Version 9): briefly, (1) normal body temperature for more than 3 days; (2) significant recovery of respiratory symptoms; (3) lung imaging showing obvious absorption and recovery of acute exudative lesions; and (4) negative results of the nucleic acid tests of respiratory pathogens for two consecutive samples (sampling interval of at least 24 h).

The term “re-positive” is used to refer to the phenomenon of a confirmed COVID-19 patient with detected SARS-CoV-2 RNA-positive post-discharge (with random test timing).

The RNA was measured by the RT-PCR method against open reading frame 1ab (ORF1ab) and nucleocapsid protein N, separately. The sequence of primers for the ORF1ab gene are as follows: Forward (5′–>3′): CCCTGTGGGTTTTACACTTAA; Reverse (5′–>3′): ACGATTGTGCATCAGCTGA. The sequence of primers for nucleocapsid protein N gene are: Forward (5′–>3′): GGGGAACTTCTCCTGCTAGAAT; Reverse (5′–>3′): CAGACATTTTGCTCTCAAGCTG. IgG or IgM antibodies against SARS-CoV-2 were detected using magnetic particle chemiluminescence, which is a highly sensitive immunoassay that can be used to determine all antigenic substances.

### Statistical analysis

Continuous variables are presented as the mean ± standard deviation for normally distributed data or median with interquartile range (IQR) for non-normally distributed data. Categorical variables are expressed as *n* (%). Differences in clinical characteristics and laboratory findings between groups were compared using the independent sample *T*-test or Mann–Whitney *U* test (continuous variables) and the chi-square test or Fisher’s exact test (categorical variables). A two-sided significance level of 0.05 was used to evaluate the statistical significance.

## Results

### Demographic features and clinical symptoms of re-positive patients

The data from 180 patients were collected in this study, of whom 118 were mild cases and 62 were asymptomatic. The patients were between 4 and 93 years of age, with an average age of 45.73 years, and 113 patients were men (62.7%). The median initial onset course was 12 days, the median course between the first COVNAT-negative and re-positive time point was 13 days, the median re-positive course was 6 days, and the median total disease course was 36 days. Fifty-three of the patients had underlying diseases (29.4%), including 18 hypertension patients, 11 diabetes patients, six cerebral infarction patients, five coronary heart disease patients, two hypotension patients, two asthma patients, two tracheitis patients, two depression patients, two pancreatitis patients, one chronic hepatitis patient, one brucellosis patient, and one arthritis patient.

### Comparison of clinical symptoms and laboratory results between first positive and re-positive period

The comparison of clinical symptoms and laboratory results between the initial onset and re-positive period is summarized in Table 1. Our results showed that the proportion of patients with symptoms during the re-positive period was significantly lower than that during the initial onset period. Moreover, we compared specific symptoms including fever, headache, nasal congestion, cough, dry throat, decreased sense of smell, fatigue, soreness of the body, diarrhea, nausea, vomiting, and poor appetite between the initial onset and the re-positive period. All proportions of the above symptoms were lower in the re-positive period than those in the initial onset period. Furthermore, the disease course was longer in the initial onset period than that in the re-positive period. Moreover, 37 patients had persistent symptoms, including cough (50%), dry or sore throat (44.7%), fatigue (13.5%), headache (13.5%), decreased sense of smell (5.2%), and fever (2.6%).

**TABLE 1:** Comparison of clinical symptoms between initial onset and re-positive period.

	Initial onset, *n* (%)	Re-positive, *n* (%)	*P*-value
Symptomatic, *n* (%)	118, 65.6	64, 35.6	<0.001
Fever, *n* (%)	64, 35.6	6, 3.3	<0.001
Headache, *n* (%)	36, 20.0	11, 6.1	<0.001
Nasal congestion or runny nose, *n* (%)	27, 15.0	4, 2.2	<0.001
Cough, *n* (%)	67, 37.2	34, 18.9	<0.001
Dry throat, *n* (%)	53, 29.4	25, 13.9	<0.001
Decreased sense of smell, *n* (%)	10, 5.6	3, 1.7	<0.001
Fatigue, *n* (%)	37, 20.6	9, 5.0	<0.001
Soreness all over the body, *n* (%)	40, 22.8	9, 5.0	<0.001
Diarrhea, nausea, vomiting, poor appetite, *n* (%)	16, 8.9	10, 5.6	<0.001
Disease duration (IQR)	12 (8, 16)	6 (3, 11)	<0.001

Differences in continuous variables were analyzed using the independent sample *T*-test or Mann–Whitney *U* test. Differences in categorical variables were calculated using Fisher’s exact or the Chi-square test as appropriate. *P*-values indicate differences between mild case and asymptomatic case groups. *P* < 0.05 was considered statistically significant.

### Comparison of clinical characteristics and laboratory results between patients with or without underlying diseases

To compare the clinical characteristics between patients with or without underlying diseases at the initial onset and re-positive period, the patients were grouped as with (*n* = 53) and without (*n* = 127) underlying diseases, respectively, and the clinical characteristics and laboratory results were compared between the two groups. Our results showed no significant differences between the two groups in terms of sex, clinical symptoms at the initial onset period, COVNAT-negative to re-positive duration, initial onset to COVNAT-negative duration, re-positive to COVNAT-negative duration, CoV O gene and N gene levels at the re-positive time, lowest CoV O gene and N gene levels during the re-positive period, anti-CoV IgM levels, and proportion of patients with elevated anti-CoV IgG and anti-CoV IgM levels. However, the patients were older, the proportion of patients with symptoms was significantly higher (at the re-positive period only), the proportion of patients who were vaccinated was lower, the total disease course was longer, and the levels of anti-CoV IgG were lower in patients with underlying diseases than those in patients without underlying diseases (Table 2).

**TABLE 2:** Comparison of clinical characteristics and laboratory results between patients with or without underlying diseases at first positive and re-positive period.

	Patients with underlying comorbidities (*n* = 53)	Patients without underlying comorbidities (*n* = 127)	*P-*value
Male, *n* (%)	35 (66)	78 (61.4)	0.614
Age, mean ± SD (years)	56.9 ± 14.6	41.4 ± 16.5	<0.001
Symptomatic at initial onset, *n* (%)	34 (64.2)	84 (66.1)	0.864
Symptomatic at re-positive, *n* (%)	27 (50.9)	37 (29.1)	0.006
Total disease course (days)	39.9 ± 9.7	34.8 ± 9.4	0.006
Vaccinated, *n* (%)	32 (60.4)	111 (87.4)	<0.001
COVNAT negative to re-positive duration, IQR (days)	11.5 (8–22)	12 (9–17)	0.647
Initial onset to COVNAT negative duration, IQR (days)	14 (9–18)	11 (8–16)	0.065
Re-positive to COVNAT negative duration, IQR (days)	6.5 (3–12.75)	6 (3–10)	0.14
CoV O gene levels at re-positive time, mean ± SD (Ct)	32.1 ± 4.9	32.6 ± 4.4	0.578
CoV N gene levels at re-positive time, mean ± SD (Ct)	30.9 ± 5.1	31.9 ± 4.1	0.478
Lowest CoV O gene levels during re-positive period, mean ± SD (Ct)	27.9 ± 5.4	29.3 ± 5.4	0.147
Lowest CoV N gene levels during re-positive period, mean ± SD (Ct)	26.5 ± 5.1	28.0 ± 5.2	0.094
Anti-CoV IgG levels at re-positive time, IQR (AU/ml)	22 (1.19–106.74)	65 (25–158)	0.001
Patient with elevated anti-CoV IgG, *n* (%)	44 (83)	116 (91.3)	0.122
Anti-CoV IgM levels at re-positive time, IQR (AU/ml)	0.38 (0–0.154)	0 (0–0.427)	0.434
Patient with elevated anti-CoV IgM, *n* (%)	8 (15.1)	16 (12.6)	0.683

CoV, coronavirus; COVID-19, coronavirus disease 2019; COVNAT, coronavirus nucleic acid test. Differences in continuous variables were analyzed using the independent sample T-test or Mann–Whitney U test. Differences in categorical variables were calculated using Fisher’s exact or the Chi-square test as appropriate. P-values indicate differences between mild case and asymptomatic case groups. P < 0.05 was considered statistically significant.

### Comparison of clinical characteristics and laboratory results between patients aged >50 and ≤50 years

To compare the clinical characteristics by age at the initial onset and re-positive period, the patients were grouped by age >50 years (*n* = 71) and ≤50 years (*n* = 109), and the clinical characteristics and laboratory results were compared between the two groups. Our results showed no significant differences between the two groups regarding sex, clinical symptoms at the initial onset period and at the re-positive period, COVNAT-negative to re-positive duration, initial onset to COVNAT-negative duration, re-positive to COVNAT-negative duration, CoV O and N gene levels at re-positive time, anti-CoV IgM levels, and proportion of patients with elevated anti-CoV IgM levels. However, the proportion of patients with underlying diseases and the proportion of non-vaccinated patients were higher, and the lowest CoV O gene Ct values, N gene Ct values, levels of anti-CoV IgG, and proportion of patients with elevated anti-CoV IgG levels were lower in patients aged >50 years (Table 3).

**TABLE 3:** Comparison of clinical characteristics and laboratory results between patients >50 and ≤50 years old.

	Age > 50 (*n* = 71)	Age ≤ 50 (*n* = 109)	*P-*value
Male, *n* (%)	47 (66.2)	66 (60.6)	0.528
With underlying disease, *n* (%)	35 (49.3)	18 (16.5)	<0.001
Symptomatic at initial onset, *n* (%)	42 (59.2)	76 (69.7)	0.152
Symptomatic at re-positive, *n* (%)	28 (39.4)	36 (33)	0.427
Total disease duration (days)	35 (31, 42)	35 (28, 43)	0.735
Vaccinated, *n* (%)	49 (69)	94 (86.2)	0.008
COVNAT negative to re-positive duration, IQR (days)	11 (8, 18)	13 (9, 18)	0.412
Initial onset to COVNAT negative duration, IQR (days)	13 (7, 17)	11 (8, 16)	0.986
Re-positive to COVNAT negative duration, IQR (days)	7 (3, 12)	5 (3, 10)	0.107
CoV O gene levels at re-positive time, mean ± SD (Ct)	32.3 ± 4.7	32.5 ± 4.5	0.796
CoV N gene levels at re-positive time, mean ± SD (Ct)	31.2 ± 4.7	31.4 ± 4.3	0.841
Lowest CoV O gene levels during re-positive period, mean ± SD (Ct)	27.6 ± 5.4	29.7 ± 5.3	0.015
Lowest CoV N gene levels during re-positive period, mean ± SD (Ct)	26.2 ± 4.9	28.5 ± 5.2	0.004
Anti-CoV IgG levels at re-positive time, IQR (AU/ml)	30.6 (1.15, 98)	78 (26, 158)	<0.001
Patient with elevated anti-CoV IgG, *n* (%)	56 (78.9)	104 (95.4)	0.001
Anti-CoV IgM levels at re-positive time, IQR (AU/ml)	0 (0, 0.43)	0 (0, 0.45)	0.984
Patient with elevated anti-CoV IgM, *n* (%)	9 (12.7)	15 (13.8)	1

CoV, coronavirus; COVID-19, coronavirus disease 2019; COVNAT, coronavirus nucleic acid test. Differences in continuous variables were analyzed using independent sample T-test or Mann–Whitney U test. Differences in categorical variables were calculated using Fisher’s exact or the Chi-square test as appropriate. P-values indicate differences between mild case and asymptomatic case groups. P < 0.05 was considered statistically significant.

### Comparison of the clinical characteristics and laboratory results between vaccinated and non-vaccinated patients

To compare the clinical characteristics between patients with or without vaccination at initial onset and in the re-positive period, the patients were grouped by vaccinated (*n* = 143) and non-vaccinated (*n* = 37) individuals, and the clinical characteristics were compared between the two groups at the first positive and re-positive periods, separately. Our results showed no significant differences between the two groups regarding sex, clinical symptoms at the initial onset period and at the re-positive period, COVNAT-negative to re-positive duration, initial onset to COVNAT-negative duration, re-positive to COVNAT-negative duration, CoV O and N gene levels at the re-positive time, lowest CoV N gene levels during the re-positive time, anti-CoV IgM levels, and proportion of patients with elevated anti-CoV IgM levels. However, the patients were older, the proportion of patients with underlying diseases was higher, and the lowest CoV O gene Ct values and levels of anti-CoV IgG, and proportion of patients with elevated anti-CoV IgG levels were lower in patients who were not vaccinated (Table 4).

**TABLE 4:** Comparison of clinical characteristics and laboratory results between vaccinated and non-vaccinated patients.

	Vaccinated (*n* = 143)	Non-vaccinated (*n* = 37)	*P-*value
Age (IQR)	44 ± 15.7	52 ± 21.47	0.006
Male, *n* (%)	89 (62.2)	24 (64.9)	0.85
With underlying disease, *n* (%)	32 (22.4)	21 (56.8)	<0.001
Symptomatic at initial onset, *n* (%)	94 (65.7)	22 (59.5)	1
Symptomatic at re-positive, *n* (%)	49 (34.3)	15(40.5)	0.564
Total disease duration (days)	34 (28, 43)	37.5 (32, 43.75)	0.052
COVNAT negative to re-positive duration, IQR (days)	12 (8, 17.75)	12 (10, 19)	0.335
Initial onset to COVNAT negative duration, IQR (days)	11 (8, 16)	13 (7, 19)	0.442
Re-positive to COVNAT negative duration, IQR (days)	6 (3, 11)	6 (3.25, 12.75)	0.345
CoV O gene levels at re-positive time, mean ± SD (Ct)	32.65 ± 4.54	31.61 ± 4.15	0.214
CoV N gene levels at re-positive time, mean ± SD (Ct)	31.62 ± 4.43	30.28 ± 4.52	0.157
Lowest CoV O gene levels during re-positive period, mean ± SD (Ct)	29.36 ± 5.4	26.89 ± 4.8	0.02
Lowest CoV N gene levels during re-positive period, mean ± SD (Ct)	27.9 ± 5.11	26.36 ± 4.89	0.119
Anti-CoV IgG levels at re-positive time, IQR (AU/ml)	79.96 (36.8, 160.07)	1.09 (0, 4.99)	<0.001
Patient with elevated anti-CoV IgG, *n* (%)	140 (97.9)	20 (54.1)	<0.001
Anti-CoV IgM levels at re-positive time, IQR (AU/ml)	0 (0, 0.486)	0 (0, 0.182)	0.239
Patient with elevated anti-CoV IgM, *n* (%)	22 (15.4)	2 (5.4)	0.173

CoV, coronavirus; COVID-19, coronavirus disease 2019; COVNAT, coronavirus nucleic acid test. Differences in continuous variables were analyzed using the independent sample T-test or Mann–Whitney U test. Differences in categorical variables were calculated using Fisher’s exact or the Chi-square test as appropriate. P-values indicate differences between mild case and asymptomatic case groups. P < 0.05 was considered statistically significant.

## Discussion

Re-positive tests for COVNAT in recovered COVID-19 patients are common, challenging the elimination of the spread of the disease. In the present study, we analyzed the data from 180 re-positive patients, including 62 asymptomatic cases and 118 mild cases, who were admitted at the First Hospital of Jilin University. Our results showed that the proportion of re-positive patients with symptoms was lower and the COVNAT-positive duration was shorter during the re-positive period. Furthermore, the proportion of patients with symptoms was higher, anti-CoV IgG levels were lower, and the total disease course was longer in patients with underlying diseases.

The results from previous studies have shown that the symptoms were generally milder in the re-positive period than those in the initial infection period (Wu et al., 2020; Li et al., 2022), most of the re-positive patients turned negative in the next few tests, and patients were found positive for antibodies against SARS-CoV-2 (Yuan et al., 2020). Accordingly, our results showed that the proportion of re-positive patients with symptoms was lower and the COVNAT-positive course was shorter during the re-positive period. The reason why symptoms were milder in the re-positive period may be due to the low viral load of re-positive patients; indeed, viral culture of samples from patients in the re-positive period showed no cytopathic effect, and nucleic acid testing of the culture medium from viral cultures showed negative results (Lu et al., 2020; Liang et al., 2021), indicating that a re-positive test is likely due to inactivated viral RNA rather than reactivation or reinfection.

Many risk factors have been reported to be associated with the severity of COVID-19, including old age; male sex; underlying comorbidities such as hypertension; diabetes; chronic lung diseases; heart, liver, and kidney diseases; obesity; tumors; clinically apparent immunodeficiencies; local immunodeficiencies; and pregnancy (Gao et al., 2021). In our cohort, 53 patients had underlying diseases (29.4%), and the proportion of patients with symptoms was higher in those with underlying diseases. In line with our findings, previous reports showed that among re-positive cases, increased underlying comorbidity was observed in re-positive patients compared to that in non-re-positive patients with COVID-19 (Guan et al., 2020; Hoang, 2021). Another study using multivariate Cox regression analysis showed that the risk factors associated with re-positivity were the presence of comorbidities, particularly hypertension or chronic diseases in the respiratory system (Zhou et al., 2020). Moreover, we found that the levels of anti-CoV IgG were lower and the total disease duration was longer in patients with underlying diseases. Interestingly, in the group with underlying diseases, the patients were older and had a lower proportion of vaccinated patients when compared with the group of patients without underlying diseases. Anti-CoV IgG is a protective IgG that is capable of adhering to the virus and preventing it from entering the host cell (Dinc et al., 2022). Vaccination is the most effective way to boost the levels of anti-CoV IgG, and our results showed that patients with underlying disease are older and less vaccinated, indicating that age and underlying disease are factors influencing the symptomatic patients and long total disease course in re-positive patients.

The patients in our cohort were between 4 and 93 years of age, with an average age of 45.73 years, and 113 patients were men (62.7%). The initial onset duration was 12 days, the time between the first COVNAT and re-positive time was 13 days, and the median total disease course was 36 days. It has been reported that re-positivity can occur in any person, but juveniles, women, and patients with mild/moderate existing symptoms have higher rates of re-positivity (Liu et al., 2021). Another study showed that patients younger than 18 years had higher re-positive rates, and no significant differences were observed regarding sex between re-positives and non-re-positives (Yuan et al., 2020). These differences may be explained by the different regions and populations included in the study. Even though pollution is mild in the northeast of China, it has recently been reported that chronic exposure to PM2.5 causes overexpression of the alveolar ACE2 receptor, the entry route of SARS-CoV-2 into the organism, shared by the lungs and testis where expression is highest in the body (Montano et al., 2021). Hence, the influence of air pollution cannot be ignored.

No significant difference in terms of clinical symptoms was found between patients aged >50 years and those aged ≤50 years. However, the proportion of patients with underlying disease were higher, the proportion of patients who were vaccinated, the lowest CoV O gene and N gene Ct values, and the levels of anti-CoV IgG were lower in patients who were older than 50 years, indicating that age, underlying disease, and vaccination are factors that influence the capacity of patients to produce protective antibodies.

Our results showed no significant difference in clinical symptoms between the vaccinated and non-vaccinated groups at either the initial onset period or the re-positive period. However, the lowest CoV O gene Ct values and the levels of anti-CoV IgG were lower in patients who were not vaccinated. The full vaccination rate was 81.1%, and the booster immunization rate was 32.1% in Jilin Province, according to the National Health Commission of China. In the present study, 81.67% of patients had the first vaccination dose, 60.5% of patients were fully vaccinated, and 18.9% of patients had a booster vaccination. Whether current vaccines are effective against the new Omicron variant of COVID-19 has been investigated: effectiveness of the vaccine against SARS-CoV-2 was lower for omicron than that for pre-omicron variants 1 month after vaccine completion, and little protection remained by 6 months after the primary vaccine series (Higdon et al., 2022). Both re-positives and non-re-positives have been reported to have similar patterns of IgG and IgM (Huang et al., 2021). Therefore, large-scale and multicenter studies are recommended to better understand the pathophysiology of the potential recurrence of SARS-CoV-2 in patients with COVID-19.

Our study had several limitations. First, it only included patients who tested re-positive for SARS-CoV-2, and no control group of non-re-positive patients was enrolled. Second, we could not differentiate between re-positive tests that were due to prolonged viral shedding of non-viable viral fragments and those that were true new infections. We should isolate samples of the live virus instead of performing RT-PCR assays to determine the true infectivity of patients for the purpose of continuous disease management.

In conclusion, recurrence of SARS-CoV-2 in patients who have recovered from COVID-19 after discharge from the hospital is common. However, the cause of this re-positivity remains unclear. Symptoms were milder, and the nucleic acid test-positive course was shorter during the re-positive period. The concomitant underlying disease is an important factor associated with clinical symptoms and the overall course of COVID-19 re-positive patients may be associated with lower anti-CoV IgG levels. Large-scale and multicenter studies are recommended to better understand the pathophysiology of the potential recurrence of SARS-CoV-2 in patients with COVID-19.

## Data availability statement

The original contributions presented in this study are included in the article/Supplementary material, further inquiries can be directed to the corresponding authors.

## Ethics statement

The studies involving human participants were reviewed and approved by Ethics Committee of the First Hospital of Jilin University. Written informed consent for participation was not required for this study in accordance with the national legislation and the institutional requirements.

## Author contributions

JC and ZW: designed the study. PZ, XY, and JN: interpreted the data. MZ: drafted the manuscript. All authors revised the manuscript critically for important intellectual content and gave final approval of the version to be published.
