# Supplementary material for: Milder symptoms and shorter course in patients with re-positive COVID-19: A cohort of 180 patients from Northeast China
Source: Front Microbiol. 2022 Oct 11;13:989879. doi: 10.3389/fmicb.2022.989879 (PMC9593080; doi:10.3389/fmicb.2022.989879)
Supplement: Supplementary file 1 [file Table_1.DOCX]

**STable 1. Comparison of clinical characteristics of re-positive COVID-19 patients between symptomatic and asymptomatic cases.**

|  | symptomatic cases（n = 118） | asymptomatic cases（n = 62） | *P* value |
| --- | --- | --- | --- |
| sex（male, female） | 70, 48 | 43, 19 | *0.199* |
| age, mean ± SD, (years) | 45.55 ± 17.67 | 46.06 ± 20.82 | *0.85* |
| total disease course, mean ± SD, (days) | 36.38 ± 10.04 | 35.39 ± 8.94 | *0.17* |
| initial onset to COVNAT negative duration, IQR (days) | 11 (7, 16) | 11 (8, 15) | *0.664* |
| COVNAT negative to re-positive duration, IQR (days) | 12 (9, 18) | 12 (9, 17) | *0.627* |
| re-positive to COVNAT negative duration, IQR (days) | 6 (3, 11) | 7 (3, 11.5) | *0.664* |
| CoV O gene levels at re-positive time, mean ± SD (Ct) | 32.34 ± 4.37 | 32.73 ± 5.00 | *0.135* |
| CoV N gene levels at re-positive time, mean ± SD (Ct) | 31.32 ± 4.15 | 31.40 ± 5.51 | *0.1* |
| lowest CoV O gene levels during re-positive period, mean ± SD (Ct) | 28.91 ± 5.44 | 29.08 ± 5.87 | *0.344* |
| lowest CoV N gene levels during re-positive period, mean ± SD (Ct) | 27.86 ± 5.40 | 27.12 ± 4.91 | *0.533* |
| anti-CoV IgG levels at re-positive time, IQR (AU/ml) | 62.5 (16.1, 150.7) | 44.5 (5, 131.42) | *0.557* |
| anti-CoV IgM levels at re-positive time, IQR (AU/ml) | 0.04 (0.00, 0.45) | 0.00 (0.00, 0.31) | *0.216* |

**Supplementary Table 1.** **Comparison of clinical characteristics of re-positive COVID-19 patients between symptomatic and asymptomatic cases.** Abbreviations: CoV = coronavirus; COVID-19 = coronavirus disease 2019; COVNAT = coronavirus nucleic acid test. Differences in continuous variables were analyzed using the Mann–Whitney *U* test. *P* values indicate differences between mild case and asymptomatic case group. *P* < 0.05 was considered statistically significant.
